# Perlecan, CollagenXVIII, and Agrin Expression in Normo‐, Hypo‐, and Aganglionic Segments in Hirschsprung's Disease

**DOI:** 10.1111/nmo.70230

**Published:** 2026-01-19

**Authors:** Nico van den Beld, Melina Fischer, Melanie Scharr, Simon Scherer, Rudi Beschorner, Bernhard Hirt, Peter H. Neckel

**Affiliations:** ^1^ Institute of Clinical Anatomy and Cell Analysis University of Tübingen Tübingen Germany; ^2^ Department of Pediatric Surgery University Children's Hospital Tübingen Tübingen Germany; ^3^ Department of Neuropathology Institute of Pathology and Neuropathology, University Hospital Tübingen Tübingen Germany

**Keywords:** enteric nervous system, extracellular matrix, Hirschsprung's disease, HSPG

## Abstract

**Background:**

Secretory heparan sulphate proteoglycans (HSPGs) interact with various morphogens, growth factors, and signaling molecules contributing to the development of the enteric nervous system (ENS). Thus, HSPGs have come into focus as pathomechanistic players of enteric neuropathies, such as Hirschsprung's disease (HSCR) in animal models. However, a detailed description of HSPG expression in human HSCR patients is missing.

**Methods:**

We characterized the expression pattern of the secretory HSPGs perlecan, COL18A1, and agrin in the human ENS and investigated differences in affected and healthy intestinal segments. Thus, comparative immunostainings were performed on human gut samples from HSCR‐patients and on non‐HSCR controls as well as tissues from human body donors.

**Key Results:**

Strikingly, we found that perlecan, COL18A1, and agrin were expressed as periganglionic basement membrane‐like structures in the human ENS. Interestingly, the expression pattern in normoganglionic and hypoganglionic HSCR‐tissues was comparable to the expression pattern in control tissues, despite the loss of neuronal differentiation markers in hypoganglionic segments. In aganglionic segments, the immunoreactivity of the investigated secretory HSPGs in the intermuscular layer was markedly reduced or not detectable. Yet, they were still readily visible in the *Tunica muscularis*, around blood vessels, and in the epithelium, with an almost unaltered immunoreactive pattern compared to the ganglionic segment.

**Conclusion:**

Our study transferred valuable findings on the role of HSPGs in ENS development gained in animal models to human HSCR patients. Beyond their implications for understanding enteric neuropathies, we discuss our findings in the context of how the extracellular matrix might regulate homeostasis and regeneration in the human ENS.

## Key Points

- Perlecan, COL18A1, and agrin are expressed in the human ENS and are part of the basement membrane ensheathing enteric ganglia.
- Expression pattern of perlecan, COL18A1, and agrin is comparable in normoganglionic segments of Hirschsprung's disease and non‐Hirschsprung controls.
- ENS‐related secretory HSPGs vanish with enteric ganglia in the transition zone from hypoganglionic to aganglionic gut segments.

## Graphical abstract

The secretory heparan sulphate proteoglycans perlecan, agrin, collagen18A1 are expressed in the periganglionic sheath of enteric ganglia in resectates of Hirschsprung's disease patients and non‐Hirschsprung controls.

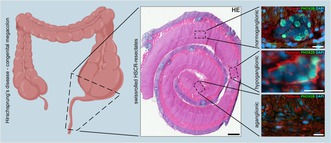

## 1 Introduction

The extracellular matrix (ECM) critically shapes organogenesis and organismic development. As a complex and dynamic structure filling the intercellular space, it interconnects cells and tissues both mechanically and via molecular signaling pathways [1].

The ECM is composed of proteins and macromolecules including glycosaminoglycans (GAGs) and proteoglycans, with a tissue‐specific composition [2, 3]. Heparan sulphate proteoglycans (HSPGs) are ECM‐molecules found throughout different developing and mature tissues with a variety of functions [4]. As glycoproteins, HSPGs consist of heparan sulphates, a subgroup of GAGs, which are covalently bound to different core proteins [5]. In mammals, HSPGs are commonly divided into three subgroups [6]: (1) transmembrane HSPGs, (2) GPI‐anchored HSPGs, and (3) secreted matrix HSPGs, namely perlecan [7], collagenXVIII (COL18A1) [8], and agrin [9]. Interestingly, HSPGs markedly influence the distribution of morphogens (e.g., Wnts) and growth factors (e.g., GDNF, FGF) in the extracellular space, thereby shaping cell–cell communication and ontogenetic processes. Recent studies also showed an involvement of secretory HSPGs in the development of the enteric nervous system (ENS) [10, 11].

The ENS is the intrinsic innervation of the gastrointestinal tract largely composed of two ganglionated plexuses: the myenteric and the submucosal plexus. During development, the ENS is formed by neural crest cells (NCCs) colonizing the gut. This process relies on properly directed cell migration, coordinated cell proliferation, and finely regulated neural differentiation [12].

Signaling pathways like GDNF‐RET [13, 14, 15, 16] and EDNRB [17, 18, 19, 20] as well as transcription factors such as PHOX2B [21] and SOX10 are crucial for proper ENS development [22, 23]. In addition, the composition of the ECM affects NCC migration and ENS formation: Structural proteins such as laminin, fibronectin, vitronectin, and collagen type I support NCC migration [24, 25], while collagen VI, versican, and collagen IX have an inhibitory effect [26, 27].

Similarly, secretory HSPGs influence NCC migration. Nagy et al. showed that NCCs secrete COL18 and agrin, whereby COL18 is highly expressed at the migrating wavefront and has a permissive effect on NCC migration [10]. In contrast, agrin mainly is expressed behind the migrating NCC wavefront, exhibiting an inhibitory effect on NCC migration, indicating that the developing ENS shapes its own microenvironment [10].

Also, perlecan is involved in proliferation and differentiation in various organ systems, possibly through the formation of morphogen gradients [1]. Perlecan/Trol stimulates the proliferation of neuroblasts in the central nervous system in *Drosophila melanogaster* [28], whereas in mice, the absence of perlecan critically affects FGF‐2 signaling in the subventricular zone (SVZ) and decreases the self‐renewal of neural stem cells and neurogenesis [29]. In contrast, interaction of perlecan with FGF‐2 stimulated neurogenesis in the SVZ [29].

HSPGs have also been shown to interact with Wnt‐ligands to facilitate downstream signaling pathways [30]. Interestingly, Wnt‐signaling was shown to be involved in ENS development [31] as well as postnatal ENS‐progenitor cell regulation [32, 33, 34, 35]. Moreover, the expression of components of the Wnt‐signaling cascade was found throughout the mature ENS [36], and was linked to Hirschsprung's disease (HSCR) in murine models [37] and HSCR‐patients [38].

Hirschsprung's disease is characterized by a reduction or absence of the ENS in distal intestinal segments. These hypo‐ or aganglionoses are caused by impaired colonization and/or failed neural differentiation of NCCs in the hindgut. The impairment of migration may be due to the failure of RET [39], GDNF [40, 41] or GFRα1 [42]. In addition to intrinsic defects of the NCCs, changes of the surrounding matrix have come into focus as aetiological factors for HSCR [43]. This is substantiated by various HSCR mouse models, which show an association with alterations in the intestinal ECM. Thus, in *Ednrb* and *Edn3* knockout mice, an increased expression of laminin, collagen IV, and GAGs in the aganglionic segment can be observed [10, 44, 45].

In the current work, we evaluated the expression of secretory HSPGs in the human ENS of HSCR patients and non‐HSCR controls. For this purpose, comparative immunostainings of secretory HSPGs in normoganglionic, hypoganglionic, and aganglionic intestinal sections were performed. Our data show that perlecan, agrin, and COL18A1 are expressed in the human intestine and form basement membrane‐like structures surrounding the ganglia of the ENS both in normo‐ and hypoganglionic gut regions, even in the absence of neuronal differentiation markers. In aganglionic segments, all three secretory HSPGs disappear in the intermuscular layer together with ENS cells, while they are still present with unchanged immunoreactivity in the surrounding non‐neural tissues. Our study provides important translational findings in unraveling the role of the ECM in HSCR, as it transfers data from recent pioneering animal studies to human patient resectates.

## 2 Material and Methods

### 2.1 Patient Samples

Samples from HSCR patients were obtained from 8 patients (2 female/6 male; 10 samples), aged 3 to 23 months (median 7 months) as detailed in Table 1. Non‐HSCR samples were collected from 9 patients (4 female/5 male), aged between 3 months and 14 years (median 7 months) as shown in Table 2.

**TABLE 1:** List of HSCR patients.

Code	Sample characteristics	Sex	Age
D1	Full thickness gut wall	♂	10 months
D2	Full thickness gut wall	♂	3 months
D3a	Mucosa and submucosa	♂	6 months
D3b	Full thickness gut wall	♂	6 months
D4a	Full thickness gut wall	♂	5 months
D4b	Mucosa and submucosa	♂	5 months
D5	Full thickness gut wall	♂	23 months
D6	Full thickness gut wall	♀	5 months
D7	Full thickness gut wall	♂	8 months
D8	Full thickness gut wall	♀	13 months

**TABLE 2:** List of non‐HSCR control cases.

Code	Sample characteristics	Sex	Age	Diagnosis
N124	Intestinal circumference	♂	4 years +7 months	Stoma reconstruction after dilation of the bowel by MMIHS^a^
N125	Intestinal circumference	♂	14 years	Intestinal duplication in the small intestine
N132	Intestinal circumference	♂	4 months	Stoma reconstruction after perforation of the bowel by meconium obstruction^b^
N137	Intestinal circumference	♂	3 months	Stoma‐reversal post NEC
N138	Intestinal circumference	♀	5 months	Stoma‐reversal post anal atresia
N147	Intestinal circumference	♀	7 months	Stoma‐reversal post NEC
N149	Intestinal circumference	♂	7 months	Intestinal atresia
N150	Intestinal circumference	♀	8 months	Stoma‐reversal post NEC
N178	Intestinal circumference	♀	1 year	Stoma‐reversal after focal intestinal perforation

^a^ We did not detect any differences in the morphology of the intestinal wall between the NEC samples and the MMIHS sample.

^b^ The patient had no diagnosis of cystic fibrosis.

All samples were collected after approval by the local ethical committee (Project Nr. 652/2019BO2 and 066/2023BO2) and with the consent of the patients' parents according to the declaration of Helsinki.

The post mortal samples were collected 15.5 h after death of a 92‐year‐old Caucasian male body donor, who died of heart insufficiency. In the latest medical record, no pre‐existing conditions of the gastrointestinal system were documented. The body donor gave his informed consent in concert with the declaration of Helsinki to use his cadaver for research purposes. The procedure was approved by the local ethical authorities (Project Nr. 362/2025BO2).

### 2.2 Histological Work‐Up of Non‐HSCR Samples and Postmortem Tissue

Before cryo‐embedding, non‐HSCR tissue samples were fixed with 4% phosphate buffered p‐formaldehyde (Merck KGaA, Darmstadt, Germany) overnight at 4°C. Then, samples were rinsed three times with phosphate‐buffered saline (PBS).

For cryoconservation, fixed samples were stored overnight in 30% sucrose solution (Applichem, Darmstadt, Germany) at 4°C. Afterwards, samples were frozen in isopentane‐nitrogen cooled TissueTek (Sakura, Staufen, Germany) and stored at −80°C until further processing. Before staining, cryosections (14–16 μm) were dried for 1 h at room temperature, following rehydration with distilled water for 30 min.

### 2.3 Pathological Work‐Up: HSCR Samples

In cases of HSCR, cross‐sections approximately 0.5 cm wide were first taken from both resection margins. The colon specimens were then further preparated using the Swiss roll technique described by Meier‐Ruge et al. [46]. Briefly, the colon segments were cut longitudinally and up to 14 cm long and approximately 1 cm wide stripes were prepared and caudo‐cranial coiled. For very long colon specimens, several swiss‐rolls were prepared. The tissue samples were frozen in a cryostat on the freezing station at approximately −45°C and cut at −20°C in 16 μm‐thick sections.

### 2.4 Immunostainings

To prevent unspecific binding of antibodies, cryosections were blocked for 30 min at room temperature with PBS containing 4% goat serum (Biochrom, Berlin, Germany) or 4% donkey serum (Biorad, Dreieich, Germany), 0.1% bovine serum albumin (Roth, Karlsruhe, Germany), and 0.1% Triton X‐100 (Roth, Karlsruhe, Germany). Then tissue sections were incubated using the following primary antibodies (Table 3). For incubation, the primary antibodies were diluted in PBS with 0.1% bovine serum albumin and 0.1% Triton X‐100 and applied overnight at 4°C in a humidity chamber.

**TABLE 3:** List of used antibodies.

	Host	Order number	Concentration	Company
*Primary antibodies*				
Perlecan	Rabbit	—	1:300	Kindly provided by Klein [47]
COL18A1	Rabbit	HPA030933	1:750	Sigma‐Aldrich, St. Louis, USA
Agrin	Goat	AF550	40 μg/mL	R&D Systems, Minneapolis, USA
DAPI (4′,6‐diamidino‐2‐phenylindol Dihydrochlorid)	—	6335	200 ng/mL	Carl Roth GmbH & Co. KG, Karlsruhe, Germany
DCX	Mice	sc‐271,390	1:20	Santa Cruz, Dallas, USA
HuC/D	Mice	A‐21271	1:50	Invitrogen, CA, USA
p75	Mice	ab3125	1:500	Abcam, Cambridge, USA
PGP9.5	Mice	7863‐1004	1:300	Bio‐RAD Laboratories, Feldkirchen, Germany
PHOX2B	Mice	66254‐1‐Ig	1:500	Proteintech, Rosemont, USA
*Secondary antibodies*				
α‐gt Alexa 546	Donkey	A11056	1:400	LifeTechnologies
α‐ms Alexa 488	Donkey	A31571	1:400	Invitrogen, CA, USA
α‐ms Alexa 488	Goat	A11029	1:400	Invitrogen, CA, USA
α‐rb Alexa 546	Goat	A11010	1:400	Invitrogen, CA, USA

Afterwards, samples were washed with PBS three times for 5 to 10 min. Secondary antibody and DAPI were diluted in PBS, 0.1% Triton X‐100, and 0.1% BSA and incubated for 90 min at room temperature (for details on secondary antibodies, see Table 3). After two washing steps with PBS for 5 min, the samples were washed in distilled water for 5 min, followed by mounting with Kaiser's glycerol gelatine (Merck, Darmstadt, Germany).

### 2.5 H&E Staining

After thawing the cryostat sections at room temperature, hematoxylin–eosin staining was performed. For this purpose, the histological sections were incubated in acidic hemalaun for 10 min at room temperature. Next, the sections were washed in distilled water and differentiated under cold running tap water for 10 min. Followed by another washing step in distilled water, the sections were incubated in non‐acidified eosin for 10 min. After a second washing step, the cryostat sections were directly mounted with Kaiser's glycerol gelatine (Merck).

### 2.6 Microscopy and Image Analysis

Images were acquired using a Zeiss Axio Imager. Z1 fluorescence microscope with Apotome module with 358, 488, 546, 647 nm for excitation and appropriate filter sets. Images were acquired using ZEN software. For better comparison between samples, the imaging modalities were kept constant throughout this study: For immunohistochemical stainings exposure time for: DAPI was 150–400 ms with 5×‐objective/30–80 ms with 10×‐objective/5–20 ms with 20×‐objective; perlecan: 200 ms with 5×‐objective/150 ms with 10×‐objective/40 ms with 20×‐objective; COL18A1: 600 ms with 5×‐objective/400 ms with 10×‐objective/60 ms with 20×‐objective; agrin: 1.5 s with 5×‐objective/600 ms with 10×‐objective/150–200 ms with 20×‐objective; neuronal or neuronal‐precursor markers 900–1500 ms with 5×‐objective/900 ms with 10×‐objective/150–400 ms with 20×‐objective. For image processing, including tiling and merging of pseudocolored immunofluorescent images, ZEISS ZEN Imaging was used. Negative controls for all secondary antibodies used are provided in Figure S6.

### 2.7 Data Mining

We reassessed previously published single‐nuclear RNA‐sequencing data by Drokhlyansky et al. [48] for the expression of secretory HSPGs. The datasets were accessed via the single cell portal of the Broad Institute (singlecell.broadinstitute.org/single_cell/) using the accession number SCP1038. Data analysis was carried out using the implemented tool.

## 3 Results

**FIGURE 1:**
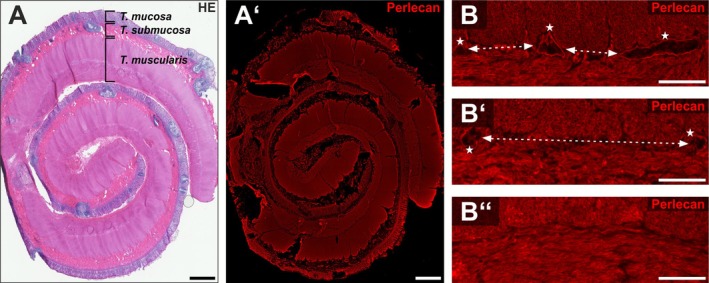
Overview of a swiss‐rolled HSCR‐resectate. An exemplary section of a swiss‐rolled gut segment stained with HE (A) and anti‐perlecan immunostaining (red) (A′). B shows representative micrographs of the intermuscular layer for a normoganglionic (B), hypoganglionic (B′) and aganglionic (B″) segment immunostained for perlecan (red). In the normoganglionic segment (B), short distances between the ganglia are observed (arrows) and the ganglia (asterisks) appear normal in size. In the hypoganglionic segments the distances between the individual ganglia become larger (B′, arrows), while the ganglia (asterisks) appear smaller. In contrast, the micrograph B″ shows an aganglionic section, where no more ganglia are detectable. Scale: A–A′: 1 mm; B–B″: 50 μm.

To evaluate the expression of secretory HSPGs in HSCR, we analyzed the expression pattern of normo‐, hypo‐, and aganglionic segments by immunohistochemistry (IHC). Intestinal resectates were rolled up and fixed using the swiss‐roll (SR) technique (Figure 1A) as part of the histopathological workflow. Since there is a gradual transition from the normo‐ (Figure 1B) to the aganglionic gut segments (Figure 1B″), we followed Meier‐Ruge et al. in defining a hypoganglionic transition zone (Figure 1B′): The area and number of ganglia of the myenteric plexus are reduced by 59% in the hypoganglionic region, while the distances between the ganglia are doubled [46]. Additionally, ganglia in the hypoganglionic segments exhibit a lower number of nerve cells per ganglion [49]. For better comparison, we defined the intermuscular layer as the location of the myenteric plexus between the *Stratum circulare* and *longitudinale* of the *Tunica muscularis*, regardless of whether it is a ganglionic or aganglionic segment.

### 3.1 Perlecan in the Normoganglionic Segment

**FIGURE 2:**
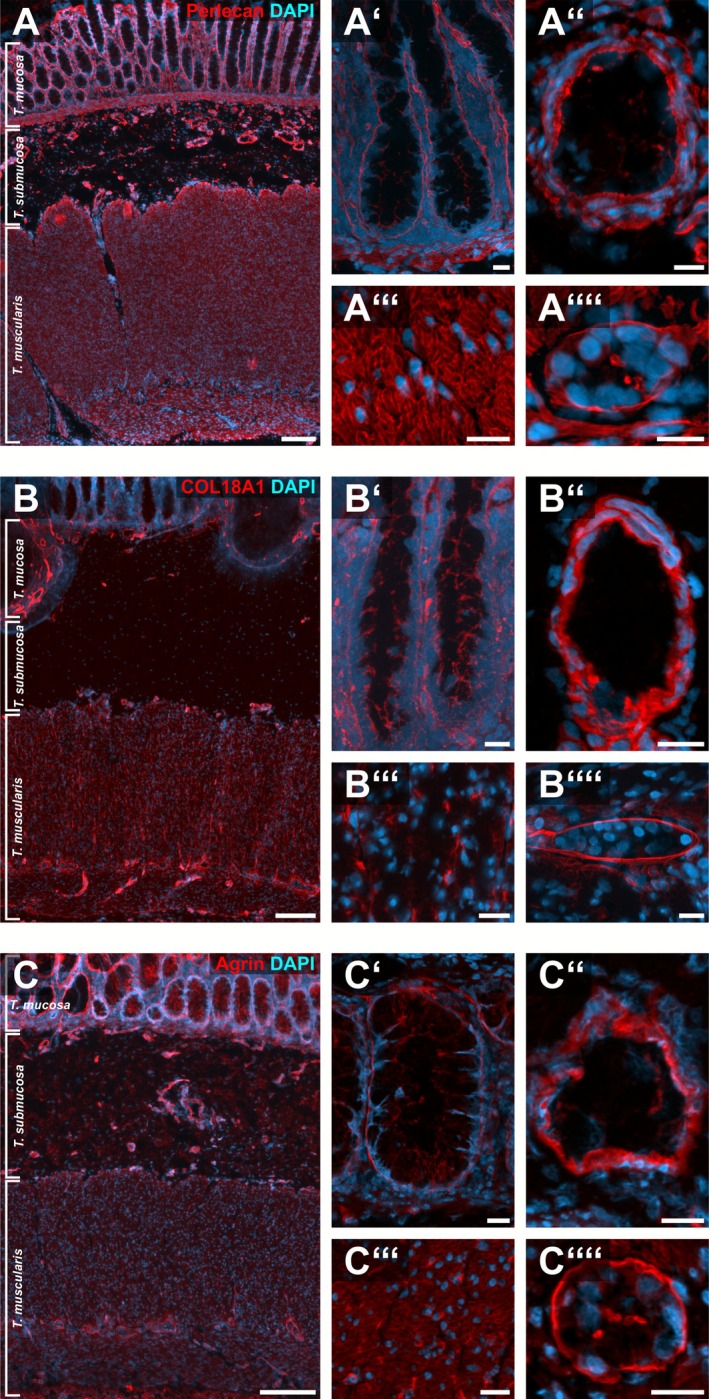
HSPG‐localization in normoganglionic colonic segments. Micrographs show immunostainings for perlecan (A), COL18A1 (B), and agrin (C) on full thickness resectates of the colon. In A′–A″″ the immunoreactivity of perlecan can be seen in high‐power magnification at the crypt base and along the crypts (A'), in the matrix of blood vessels (A″) and in the matrix of smooth muscle cells of the *Tunica muscularis* (A‴). A fine layer of perlecan was readily detectable in the basement membrane surrounding myenteric ganglia in the intermuscular layer (A″″). Comparably, COL18A1 and agrin immunoreactivity was found along the entire crypt (B′ and C′) and in the matrix of the blood vessels (B″ and C″). However, we did not detect a honeycomb‐like staining for COL18A1 in the *Tunica muscularis* (B‴) as it was found for perlecan (A‴) or agrin (C‴). Yet, the basement membrane of myenteric ganglia was clearly immunoreactive for COL18A1 (B⁗) and agrin (C⁗). Cell nuclei were counterstained with DAPI. Scale: A–C: 200 μm; A′–A⁗/B′–B⁗/C′–C⁗: 20 μm.

Within the normoganglionic region of HSCR‐affected colon resectates (see also Figure 1B′), we detected a strong immunoreactivity for perlecan in the matrix surrounding smooth muscle cells of the *Tunica muscularis* (Figure 2A‴) as well as in submucosal blood vessels (Figure 2A″). Moreover, we found strong perlecan signals in the epithelial basement membrane throughout the intestinal crypts (Figure 2A′).

**FIGURE 3:**
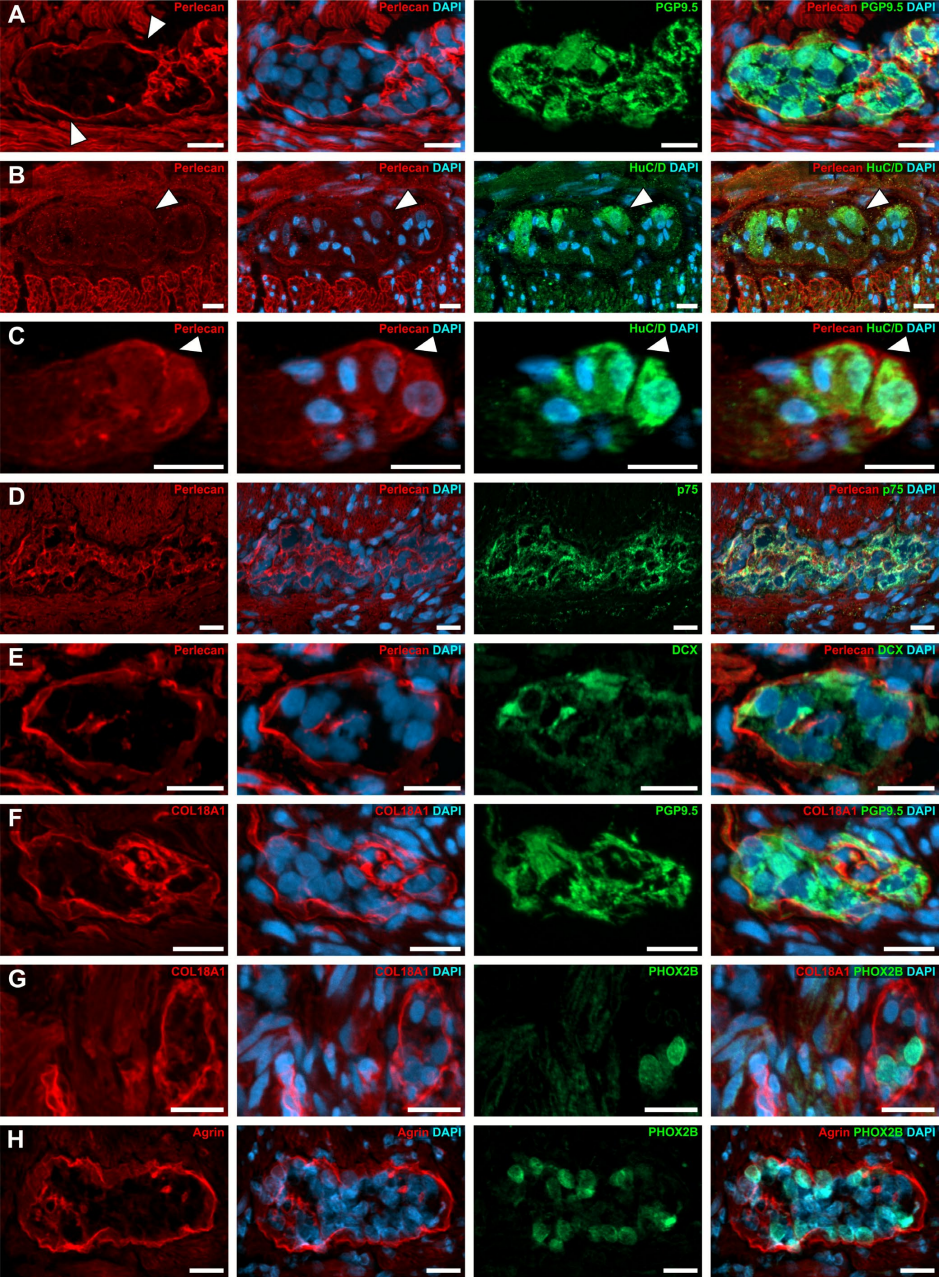
Expression of perlecan, COL18A1, and agrin around enteric ganglia in the normoganglionic segment of a HSCR affected human colon. In the normoganglionic colon perlecan expression was detectable in the matrix (arrowheads) surrounding PGP9.5 positive myenteric ganglia (A) as well as myenteric (B) and submucosal (C) ganglia with HuC/D positive perikaryal. Moreover, we detected pan‐neural markers (p75, D), as well as developmental markers DCX (E) and PHOX2B (G + H) in ganglia of normoganglionic segments. Furthermore, a strong staining for COL18A1 (F + G) and agrin (H) was detectable as a fine layer around myenteric ganglia. In some cases, a weaker, homogeneous immunopositivity of perlecan (B + C), COL18A1 (G) and agrin (H) was found inside the ganglia. Cell nuclei were counterstained with DAPI. Scale A–H: 20 μm.

Additionally, we detected a fine, intensely stained perlecan layer as part of the basement membrane surrounding submucosal (Figures 3C and S1A) and myenteric ganglia of the ENS (Figures 2A‴ and 3A,B,D).

In some ganglia, we found a considerably stronger perlecan immunoreactivity in parts of the ganglionic basement membrane (Figure 3A, arrowheads) that made direct contract to a HuC/D‐positive perikaryon (Figure 3B,C, arrowheads). This suggests that these neurons might directly or indirectly influence the composition of the adjacent ganglionic matrix envelope (Figure 3A–C). However, this observation was not persistent throughout all ganglia and samples (Figure 3B vs. C). It is also noteworthy, that we occasionally found a weak and homogeneous intraganglionic staining for perlecan (Figure 3B,C).

### 3.2 COL18A1 in the Normoganglionic Segment

We also found a robust immunoreactivity for COL18A1 in the normoganglionic colonic segments of HSCR patients. In contrast to perlecan, we did not detect COL18A1 in the musculature of the *Tunica muscularis* (Figure 2B).

Instead, COL18A1 was only present surrounding blood vessels (Figure 2B″) and nerves within the *Tunica muscularis* (Figure 2B‴). Moreover, COL18A1 was well distinguishable in the epithelial basement membrane at the base of the crypts and along the crypts (Figure 2B′).

Concerning the ENS, we found an intense staining of COL18A1 as a fine, basement membrane‐like layer surrounding submucosal and myenteric ganglia (Figures 3F,G and S1B). This fluorescence was equally strong in both plexuses. Occasionally, it is also present within the ganglion in a lower intensity (Figure 3G).

### 3.3 Agrin in the Normoganglionic Segment

Agrin exhibited a strong signal surrounding individual muscle cells of the *Tunica muscularis* forming a honeycomb pattern; however, less crisp compared to perlecan (Figure 2C‴). In addition, we found an intense staining surrounding blood vessels (Figure 2C″) and along the crypts comparable to perlecan (Figure 2C′).

In the ENS, we also detected a strong immunoreactivity of agrin surrounding the ganglia of the submucosal and myenteric plexus resembling a basement membrane (Figures 3H and S1C). In some cases, a weak homogeneous immunopositivity of agrin was also found inside the ganglia.

As expected, the expression patterns of perlecan, COL18A1, and agrin in the normoganglionic segment of HSCR patients were comparable to colonic tissues from pediatric non‐HSCR patients (Figure S2A: perlecan; B: COL18A1; C: agrin).

To assess if HSPG‐expression is specific to the young gut and might change later in life, we evaluated small intestinal samples from a human body donor, aged 92, without preexisting gastroenterological diagnoses. Interestingly, we found a similar expression pattern of Perlecan (Figure S3A), COL18A1 (Figure 3B), and agrin (Figure S3C) in the basement membrane around vessels (Figure S3A″,B″,C″), muscles (Figure S3A‴,B‴,C‴), crypts (Figure S3A′,B′,C′), and enteric ganglia (Figure S3A⁗,B⁗,C⁗), in these postmortem tissues comparable to the pediatric patients, indicating that HSPGs are stable components of the intestinal matrix throughout life.

### 3.4 Use of Developmental Markers to Facilitate Comparative Analysis Across Normo‐, Hypo‐ and Aganglionic Segments

Since hypo‐ and aganglionic gut segments often lack neuronal differentiation markers despite the presence of ENS‐cells [50], we also included markers of immature neural cells to facilitate the comparative analysis of ENS‐related HPSG expression throughout affected and unaffected gut regions. Therefore, we stained for the pan‐neural neurotrophin‐receptor p75, the transcription factor PHOX2B, as well as doublecortin (DCX), a microtubule‐associated protein expressed by neural precursors and immature neurons in the CNS.

**FIGURE 4:**
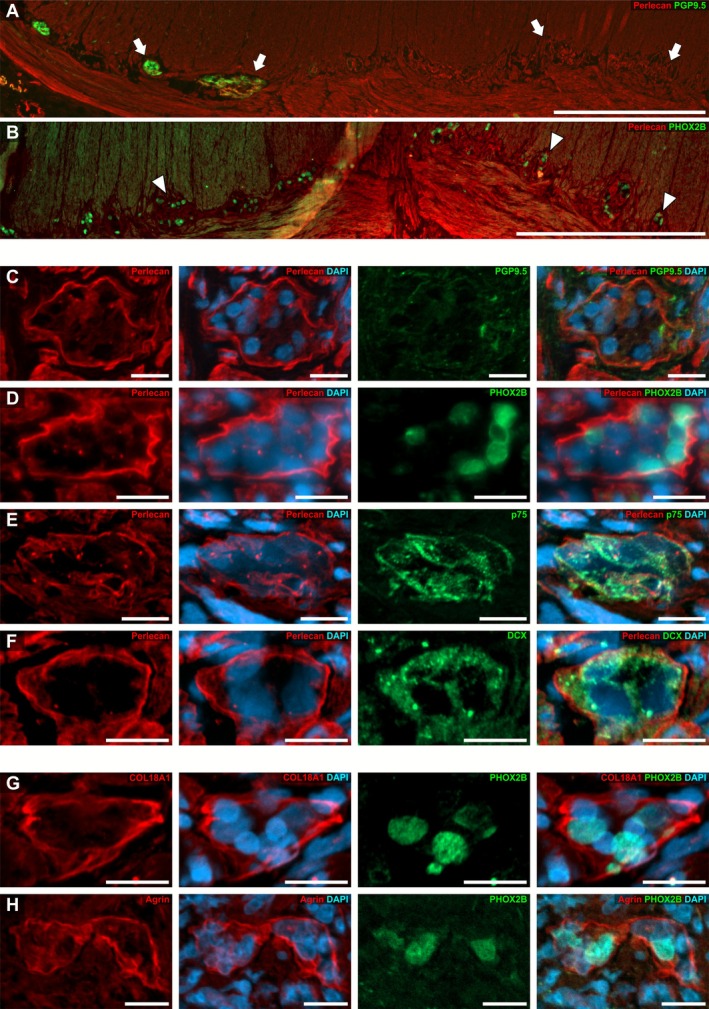
Expression of perlecan, COL18A1, and agrin in the hypoganglionic segment. The overview of the *Tunica muscularis* at the transition zone from normo‐ to hypoganglionic gut region in HSCR showed an abrupt vanishing of immunoreactivity for the neuronal marker PGP9.5 (A, arrows), whereas PHOX2B remained unchanged throughout hypoganglionic ganglia stained on a consecutive section of the same patient (B, arrowheads). (C–H) Show high‐power magnification micrographs of myenteric ganglia in the hypoganglionic region. The expression pattern of perlecan remained unchanged in comparison to the normoganglionic segment, while PGP9.5 became undetectable (C). In contrast, PHOX2B (D), as well as p75 (E) and DCX (F) were readily visible in hypoganglionic ganglia. Similar to perlecan, the expression pattern of COL18A1 (G) and agrin (H) was unchanged compared to the normoganglionic segment and surrounded the myenteric ganglia resembling a basal membrane. Cell nuclei were counterstained with DAPI. Scale: A + B: 200 μm, C–H: 20 μm.

Within the normoganglionic segment, we found p75 (Figure 3D) and PHOX2B (Figure 3G,H) immunostainings in line with previous reports [50] (Figures 3 and 4). PHOX2B is particularly useful in identifying ganglion cells due to its nuclear localization and was thus used for most experiments (Figure 4B). Moreover, DCX was well detectable in the normoganglionic ENS (Figure 3E), however, it is yet to be elucidated if it serves comparable functions as in the CNS.

### 3.5 Perlecan, COL18A1, and Agrin in the Hypoganglionic Segment

To evaluate potential changes of the HSPG expression in hypo‐ and aganglionic gut segments, we compared the morphology as well as fluorescent pattern and intensity in swiss‐rolled HSCR‐resectates including normo‐, hypo‐, and aganglionic regions.

Generally, we found that the number and size of ganglia were considerably reduced in the transition zone from ganglionic to aganglionic gut segments. Occasionally, we also detected ectopic ganglia located within one of the muscle layers of the *Tunica muscularis*.

In the transition from the ganglionic to the hypoganglionic segment, the expression of the mature neuronal marker PGP9.5 vanished abruptly (Figure 4A). Interestingly, the immunofluorescence signal of PHOX2B (Figure 4B) as well as the signal of the immature neuronal markers p75 (Figure 4E) and DCX (Figure 4F) remained unchanged in intensity in these PGP9.5 negative ganglia of the hypoganglionic region (Figure 4C–E). Intriguingly, we found that perlecan (Figure 4A–F), COL18A1 (Figure 4G), and agrin (Figure 4H) in hypoganglionic gut regions exhibited a strong and defined immunoreactivity surrounding the enteric ganglia in a comparable way as in the normoganglionic gut segments (see Figure 3A,F,H).

### 3.6 Perlecan, COL18A1, and Agrin in the Aganglionic Segment

**FIGURE 5:**
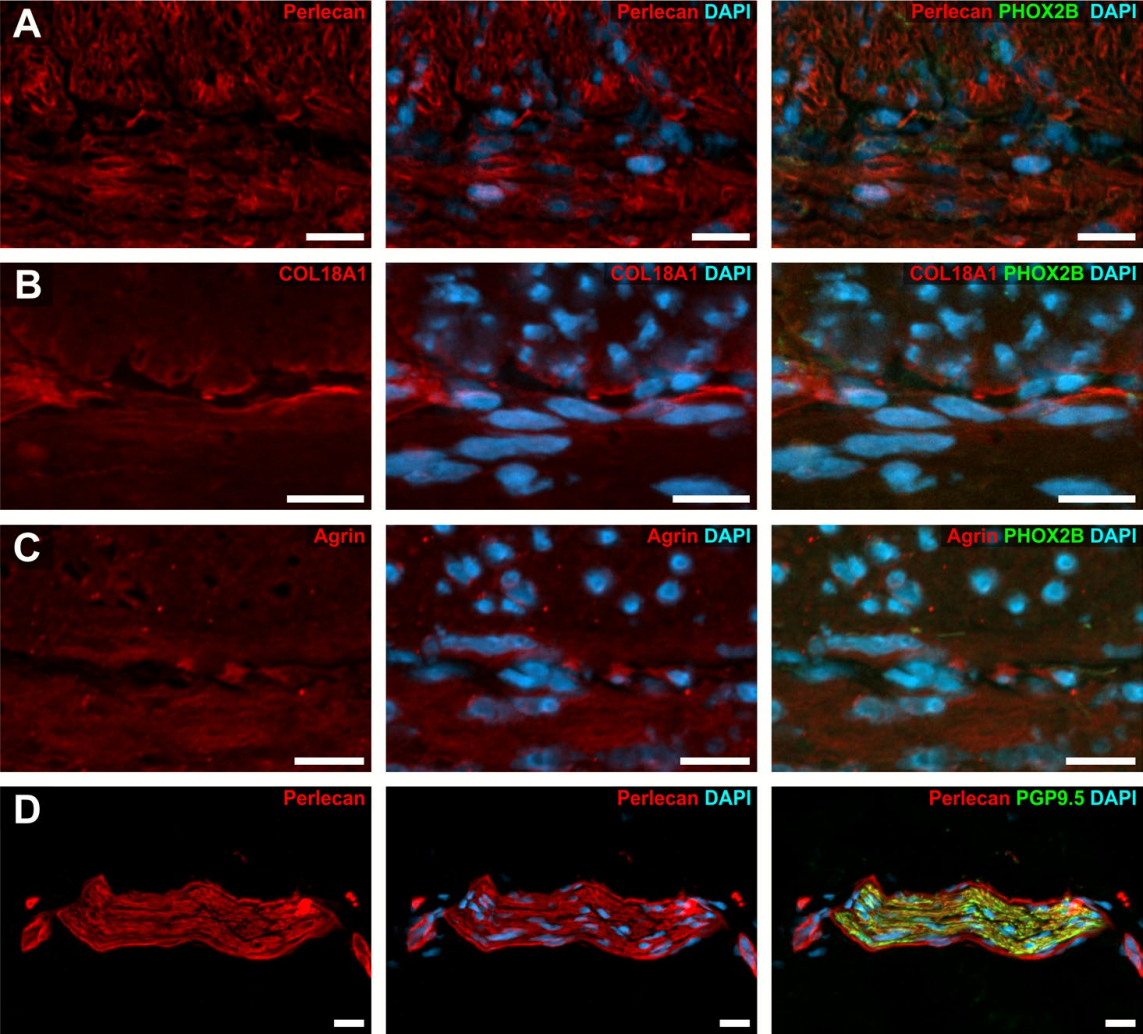
Expression of perlecan, COL18A1 and agrin in the aganglionic segment. The micrographs in A–C show the layer between the longitudinal and circular muscle layers in the aganglionic gut region immunostained for perlecan, COL18A1, and agrin. While perlecan (A) and COL18A1 (B) were both markedly reduced and partly absent in the fine connective tissue separating the muscle layers, only perlecan was clearly visible throughout the smooth musculature. In contrast, agrin (C) was detectable in the *Tunica muscularis*; however, with a less defined immunoreactivity resembling the honeycomb pattern as in the ganglionic regions. Moreover, agrin was hardly detectable in the intermuscular layer of the aganglionic segments. Additionally, we found that extrinsic nerves exhibited a crisp immunoreactivity for perlecan (D). Cell nuclei were counterstained with DAPI. Scale A–D: 20 μm.

In the aganglionic segment, perlecan (Figure 5A), COL18A1 (Figure 5B), or agrin (Figure 5C) was absent or hardly detectable in the intermuscular layer. In contrast, their individual expression pattern and occurrence in the surrounding tissue (i.e., musculature, blood vessels, or mucosa (Suppl. Figure S4A–C)) remained unchanged. Compared to normo‐ and hypoganglionic gut regions, the circular and longitudinal muscle layers were separated only by a thin sheet of connective tissue in the aganglionic gut segments (Figure 5A–C). We also found hypertrophic PGP9.5‐positive extrinsic nerve bundles, which are frequently described in these segments in association with Hirschsprung's disease, exhibiting a distinct immunofluorescence for perlecan (Figure 5D).

To provide further insight, Figure S5 shows a side‐by‐side comparison of secretory HSPGs in the intermuscular layer of normo‐, hypo‐, and aganglionic segments in HSCR. This particularly highlights the lack of perlecan, COL18A1, and agrin in the intermuscular layer of aganglionic gut regions. Additionally, except for the missing immunoreactivity for HPSGs in the intermuscular layer in aganglionic segments, we found that the overall fluorescent intensities of the gut wall for perlecan (Figure S4A′–A‴), COL18A1 (Figure S4B′–B‴), and agrin (Figure S4C′–C‴) remained equally strong throughout the entire swiss‐roll. This suggests that the vanishing of the signal in the intermuscular layer of aganglionic segments was indeed due to a decreasing expression of the respective HSPGs surrounding myenteric ganglia rather than to an artificial gradient of the staining procedure (Figure S4).

### 3.7 Expression of Secretory HSPGs in Single Nuclear Sequencing Data of the Human Colon

**FIGURE 6:**
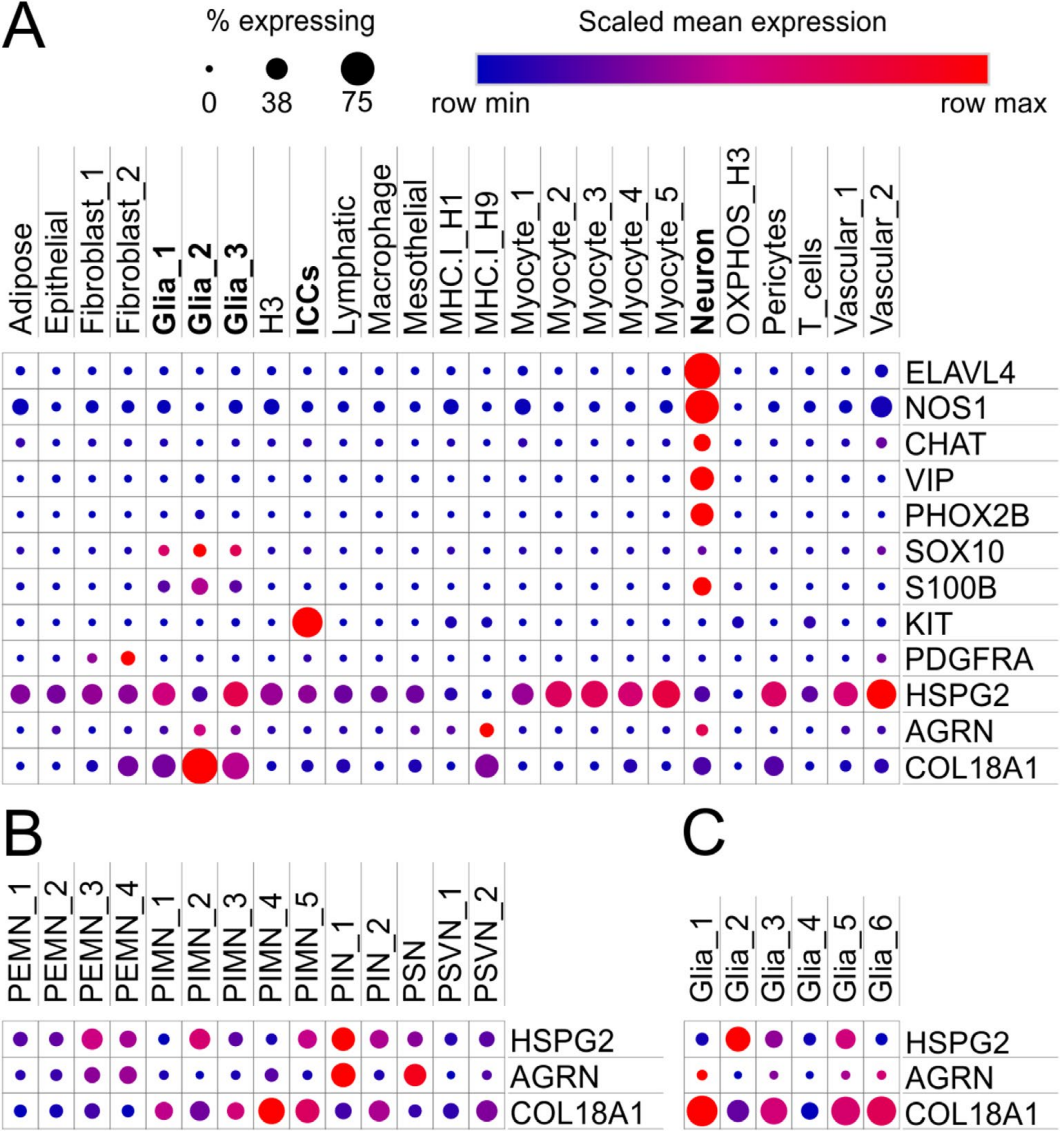
Expression of secretory HSPGs in the human colon on single‐cell resolution. The graphic shows the fraction of cells (dot size) in different annotated cell clusters (columns) expressing selected genes (rows) that are enriched in the human colon. The mean levels in expressing cells are visualized by the dot color. In (A), the expression of neuronal, glial, and pacemaker marker genes along with perlecan (HSPG2), COL18A1, and agrin (AGRN) in different cell clusters is shown. In (B) and (C), the expression of secretory HSPG genes in neuronal and glial subtypes as defined by Drokhlyansky et al. is shown (PEMN, putative excitatory motoneuron; PIMN, putative inhibitory motoneuron; PIN, putative interneuron; PSN, putative sensory neuron; PSVN, putative secretomotor/vasodilator neuron). The dataset is based on Drokhlyansky et al. [48].

To identify the cell types secreting HSPGs, we reanalyzed droplet‐based MIRACL‐Seq data of the human colon published by Drokhlyansky et al. [48]. As shown in Figure 6 and in line with our immunostainings, we found a broad expression of perlecan (HSPG2) throughout various cell types in the human colon, including enteric glial cells, myocytes, and endothelial cells, and less so in enteric neurons and pacemaker cells (ICCs). COL18A1 was found particularly in enteric glial cell clusters, as well as enteric neurons and PDGFRA‐expressing fibroblasts clusters (i.e., putative fibroblast‐like cells), whereas agrin expression was more confined to neuronal and glial subclusters.

Interestingly, we found that secretory HSPGs exhibited differential expression patterns in different neuronal subtypes: thus, the expression of perlecan (HSPG2) and agrin were more similar and particularly expressed in an interneuron subcluster (PIN_1), sensory neurons (PSN), and two excitatory motoneuron clusters (PEMN_3, PEMN_4), whereas COL18A1 was found throughout inhibitory motoneuron clusters or secretomotor/vasodilator neurons (PSVN2). In glial cells, particularly COL18A1 and perlecan (HSPG2) were expressed.

This suggests that periganglionic sheaths of HSPGs are likely produced by ENS cells themselves, although a secretion of directly adjacent ICCs and fibroblast‐like cells cannot be excluded for perlecan and COL18A1.

## 4 Discussion

Heparan sulphate proteoglycans (HSPGs) interact with various morphogens, growth factors, and signaling molecules thereby acting as diverse extracellular modulators in organogenesis and cell signaling. Nagy et al. demonstrated modulatory properties of secretory HSPGs during ENS development in the chicken model [10, 11] making them key players for a deeper understanding of enteric neuropathies, such as Hirschsprung's disease (HSCR). However, previous studies did not cover the expression pattern of HSPGs in enteric neuropathies in the human gut.

Here, we mapped the expression of the secretory HSPGs perlecan, COL18A1, and agrin in the human intestine of HSCR patients and non‐HSCR controls, particularly focusing on the ENS. We found that secretory HSPGs are an abundant component of the basement membrane surrounding myenteric and submucosal ganglia both in the juvenile and adult ENS. Using resectates from HSCR patients, we also verified the periganglionic expression of these HSPGs in the normo‐ and hypoganglionic gut segments, whereas in the intermuscular layer of aganglionic gut regions their expression was markedly reduced.

### 4.1 HSPGs in the Periganglionic Matrix—Shaping the Niche?

The expression of HSPGs in the matrix enveloping the enteric ganglia is particularly interesting considering their ability to bind and potentiate the biological activity of various morphogens and growth factors including Wnt molecules [30, 51] and fibroblast‐like growth factors [37].

Although the mechanisms underlying particularly the Wnt‐potentiating effects are not fully understood, several models have been proposed: Thus, HSPGs could act as co‐receptors, directly facilitating Wnt‐Frizzled complex formation [52, 53], or could stabilize Wnt‐proteins, thereby protecting them from degradation and contributing to the local accumulation of Wnt‐proteins [52, 54, 55]. Accordingly, a higher number of Wnt ligands would be available locally for Frizzled receptors binding [52].

Since bioactive Wnt‐molecules are hydrophobic due to posttranslational palmitoylation essential for signaling activity [56], it remains unclear how they build up long‐distance morphogen‐gradients throughout the hydrophilic extracellular space. Thus, the transport by lipoprotein particles, exosomes, or interaction with ECM‐molecules, including HSPGs, was proposed [57, 58]. Indeed, in *Drosophila*, the HSPG‐family of glypicans was found to create hydrophobic compartments by conformation changes that bind and protect the lipid‐residues of Wnt‐proteins thus supporting the establishment and maintenance of Wnt‐gradient [59]. Conceivably, perlecan as well as COL18A1 and agrin could play a similar role in binding and distributing Wnt‐molecules throughout the extracellular space. In fact, several studies indicate that perlecan and COL18A1 indeed bind Wnts [60, 61], often also restricting Wnt activity to concise areas within tissues. Intriguingly, while previous reports showed a modulatory influence of the Wnt‐regulatory network on the proliferation and differentiation of ENS cells [32, 34, 62], we recently reported that several Wnt‐ligands are expressed by neurons and/or enteric glial cells within the ganglia [36], which together with the periganglionic HSPG expression presented here might suggest an independent intraganglionic Wnt‐niche shaping cellular homeostasis of the ENS.

Additionally, beyond the function of the core protein, also the posttranslational modifications, that is, the heparan sulfate and chondroitin sulfate chains play an essential role in interacting with various morphogens including Wnt [63], FGF [64], or GDNF [65]. For the perlecan‐FGF interaction in particular, previous studies indicated an ambivalent influence of the side chains on the signaling outcome: While heparan sulfate chains induced signaling activity by facilitating FGF‐FGFR interaction, chondroitin sulfate chains physically inhibit FGF from binding to its receptor resulting in a negative regulation of the pathway (reviewed in detail by [1]). This suggests that HSPGs could serve as interactive matrix components balancing proliferation and differentiation in development, regeneration, or tissue homeostasis.

This very notion is supported by our published microarray data on cultured murine ENS progenitor cells: During experimentally induced differentiation of murine enterospheres, we observed a significant upregulation of perlecan and COL18A1 expression in the early differentiation phase [66]. This suggests that the expression of HSPGs might be part of enteric neural differentiation processes or gangliogenesis. Additionally, in the CNS, pretreatment of dopaminergic neuronal progenitors in a Parkinson's disease model with perlecan‐conjugated laminin E8 fragments resulted in enhanced maturation and neurite extension of progenitor cells through activation of BDNF–TrkB and GDNF–RET signaling [67]. The latter is also required for proper NCC migration and for the formation of neuron extension in the ENS [15].

Notably, perlecan can interact with BMPs [51, 68], which have pleiotropic effects on ENS development by co‐regulating NCC migration and differentiation into neurons and glia as well as supporting gangliogenesis [69, 70, 71]. Additionally, BMPs have a regulatory effect on the expression of the developmental transcription factor PHOX2B [72], which is involved in enteric neuron differentiation. Interestingly, BMP2 and BMP4 promote the aggregation of developing enteric neurons in vitro, which contributes to ganglion‐like clusters [73]. Noteworthy, BMP2 can bind to perlecan's domain I and there is evidence that perlecan significantly promotes its activity in vitro [68]. This supports our hypothesis that perlecan is not only a mere structural surrounding of the ganglionic niche but actively influences the formation and shape of enteric ganglia.

### 4.2 HSPGs in Hirschsprung's Disease

Given these modulatory effects of the extracellular matrix on developmental programs, changes of HSPG expression are currently getting more into focus in elucidating the underlying pathomechanisms of HSCR and other enteric neuropathies. Interestingly, Nagy et al. showed COL18A1 and agrin were clearly concentrated as a periganglionic sheath in the developing chick intestine and disappeared in aganglionic segments, while perlecan was rather expressed by the surrounding musculature [10]. Our observations support this finding as both agrin and COL18A1 were detectable encircling the enteric ganglia in normo‐ and hypoganglionic regions and essentially vanished in the layer between the muscle sheets in the aganglionic HSCR segment in human patients. Intriguingly, however, other than the findings by Nagy et al., we found that perlecan was also clearly forming the periganglionic surrounding of submucosal and myenteric ganglia in normo‐ and hypoganglionic gut regions. This discrepancy could possibly be explained by the size difference between the chicken/mouse and the human gut wall: In smaller animals, the distance between the smooth muscle cells and the myenteric ganglia is smaller than in larger mammals. Thus, in small animals perlecan expressed by the surrounding musculature might be sufficient to participate in a ganglionic sheath, whereas the larger distance between the musculature and the ganglion in humans demands an additional perlecan‐expression by the ganglion cells themselves. Furthermore, since Nagy et al. used prenatal tissues, whereas we employed postnatal patient samples, we cannot exclude that perlecan expression changes perinatally, thus contributing to these differences.

Additionally, our data mining approach of single‐cell RNA‐expression data of the human intestine published by Drokhlyansky et al. [48] strongly suggests that enteric neurons and glial cells express secretory HSPGs. Although these data were collected from an adult patient collective, these findings are generally in line with the secretion of COL18A1 and agrin in the fetal and early postnatal ENS‐cells in mice [10]. Additionally, our analysis showed a differential expression of HSPGs in different neuronal subtypes suggesting that the composition and modulation of the surrounding ECM might both influence and be part of neuronal differentiation profiles. This might also explain our inconsistent finding that the perlecan‐immunoreactivity of the ganglionic sheath often was considerably stronger if a neuronal perikaryon was directly contacting it (Figure 3A). Indeed, a work by Raghavan and Bitar supports the notion that enteric neuron subdifferentiation is influenced by matrix components in a tissue engineering approach of innervated smooth muscle sheets [74]. Nevertheless, our analysis also showed that other cell types are involved in the expression of HSPGs, particularly perlecan. Since myogenic pacemaker cells (i.e., ICCs and fibroblast‐like cells) are in very close contact to the myenteric ganglia, their contribution to the composition of the periganglionic sheath cannot be excluded and appears rather conceivable.

During development, the establishment and composition of the periganglionic sheath is largely unknown. In chickens, COL18A1 is detectable in the periganglionic envelope of the mid‐ and hindgut from E7 onwards, while agrin occurs at E10 in the proximal hindgut and is only fully expressed up to the distal hindgut after the complete colonization with ENCCs by E12 and parallels the consolidation of ganglia [10]. In addition, in mice, the density of collagen I fibers in the myenteric plexus layer increases during the formation of the plexus between E14.5 and E17.5, arguably contributing to the proper orientation of ENCCs in the ganglion compartment [75] suggesting that the ECM interacts with the developing ENS.

Indeed, recent single cell sequencing data from HSCR patients showed an upregulation of matrix components including collagens, fibronectin, and laminins in intestinal stroma and glial cells along with an increased number of fibroblasts and myofibroblasts in aganglionic gut segments [76]. The changing composition of the extracellular matrix, however, is critical for proper ENS development in that it renders the mesenchyme permissive for NCC migration and later induces neural differentiation. Thus, the delay of migratory movements along the gut might induce maldifferentiation of NCCs as the local environment is too mature already to support normal differentiation of late arriving NCCs (reviewed in more detail by [24]).

Nevertheless, NCCs are not just a product of the extracellular circumstances, but instead actively participate in shaping their own matrix environment. Thus, NCCs secrete matrix metalloproteinases, predominantly via MMP‐2 [10, 77, 78] and matrix components including agrin and COL18A1 [10]. Additionally, it is also noteworthy that the periganglionic matrix is proposed to have a barrier function and that break down of this barrier, such as in inflammatory bowel disease, might result in impaired homeostasis of the intraganglionic compartment [79].

### 4.3 Control of NCC‐Migration as a Target for Future Cell‐Based Therapies

Agrin in particular is currently coming into focus as a modulatory approach for future cell‐based therapies for HSCR [10]. Our results confirm a periganglionic expression of agrin in the human ENS, as previously observed in chicken and mouse models [10]. Agrin is expressed by trailing NCCs that have completed their migration and initiated neuroglial differentiation as well as by cells in the surrounding matrix. During this process, agrin has an inhibitory effect on NCC migration [11, 80]. This is important because blocking agrin leads to increased NCC migration in vitro, as demonstrated by Mueller et al. in enteric neurospheres of adult Wnt1; tdT mice [11]. This led the authors to hypothesize that blocking agrin could play a role in future stem cell therapies for aganglionoses, such as HSCR, by increasing cell migration after transplantation into the affected intestinal segment [11].

Notably, several matrix components including agrin, perlecan, COL18A1, as well as MMP‐2 are also expressed in murine enterospheres [66], thereby rendering these in vitro models useful tools for future studies on the influence of the extracellular matrix in ENS homeostasis and potential therapy approaches.

### 4.4 The Significance of Immunohistochemical Staining of Secretory HSPGs in the Clinical Diagnosis of Hirschsprung's Disease for Histopathology

Currently, acetylcholinesterase staining is the “gold‐standard” for intraoperative Hirschsprung's diagnosis [81]. Immunohistochemical staining for calretinin is also considered an alternative or supplement [82]. Thus, co‐staining with perlecan, COL18A1, or agrin might be helpful for reliable ganglion identification. COL18A1 appears the most suitable candidate of these three secretory HSPGs for ensuring rapid ganglion identification due to its strong staining around the ganglia with comparatively low signal activity in the *Tunica muscularis*. Beyond HSCR, immunohistochemical stainings for perlecan, COL18A1, and agrin could be of diagnostic value in desmosis coli, which is characterized by a disturbed ECM architecture [83]. However, there are currently no studies examining a connection between desmosis coli and an impaired HSPG structure.

## 5 Conclusion

In summary, secretory HSPGs appear to influence and modulate ENS development in many ways by interacting with diverse morphogens, growth factors, and signaling molecules and by affecting the composition of the periganglionic matrix. In this study we demonstrated that the secretory HSPGs perlecan, COL18A1, as well as agrin are expressed in the human intestine, and particularly around enteric ganglia. This suggests that respective observations on secretory HSPGs in rodent, *Drosophila*, and chicken models are likely transferable to the development of the human ENS. However, their effect and function on development and cellular homeostasis in the ENS have not yet been sufficiently investigated. Hence, future studies should continue to focus on the modulatory and regulatory properties of secretory HSPGs especially on the ENS.

## Author Contributions

Nico van den Beld: acquisition of data; analysis and interpretation of data; drafting of the manuscript; critical revision of the manuscript for important intellectual content. Melanie Scharr, Melina Fischer, Simon Scherer, Bernhard Hirt: analysis and interpretation of data; critical revision of the manuscript for important intellectual content. Rudi Beschorner: acquisition of data; critical revision of the manuscript for important intellectual content. Peter H. Neckel: study concept and design; acquisition of data; analysis and interpretation of data; drafting of the manuscript; critical revision of the manuscript for important intellectual content; study supervision.

## Funding

The project was supported by a grant from the German Research Foundation (DFG, Grants 438504601 and 554598887) and by intramural funding by the Medical Faculty, University Tübingen (IZKF, Grant 9748903E.05.00796).

## Conflicts of Interest

The authors declare no conflicts of interest.

## Supporting information


**Figure S1:** Representative submucosal ganglia in the normoganglionic segment. The micrographs show immunostainings for perlecan (A), COL18A1 (B), and agrin (C) in submucosal ganglia of the normoganglionic segment in HSCR‐patients. Neural cells were counterstained with PHOX2B, cell nuclei were stained with DAPI. Scale: 10 μm.

**Figure S2:** Representative transversal sections of human non‐HSCR control small intestinal segments with perlecan, COL18A1, and agrin staining. The overviews show the entire gut wall immunostained for perlecan (A), COL18A1 (B), and agrin (C). The expressions of perlecan, COL18A1, and agrin were visible at the crypt base and along the crypts, as well as in the surrounding matrix of blood vessels. Furthermore, perlecan and agrin were detectable in the surrounding matrix of smooth muscle cells of the *Tunica muscularis*, which was not the case for COL18A1. The inserts in A–C are high power magnifications of myenteric ganglia showing that a fine layer of perlecan, COL18A1, and agrin can be seen surrounding the enteric ganglia resembling a basement membrane (arrowheads). Of note, the insert in A is taken from a subsequent section than the overview. Cell nuclei were counterstained with DAPI. Scale: A–C: 200 μm; inserts: 50 μm.

**Figure S3:** HSPG expression in human non‐HSCR postmortem tissue. Depicted are overviews of the entire gut wall immunostained for perlecan (A), COL18A1 (B) and agrin (C). The high‐power magnification micrographs show myenteric ganglia (A'–C′), submucosal ganglia (A″–C″), blood vessels (A‴–C‴), and the mucosa (A⁗–C⁗). Comparable to pediatric samples, immunoreactivity of perlecan (A′), COL18A1 (B′), and agrin (C′) was detectable surrounding the myenteric ganglia. However, particularly stainings of the mucosal epithelium and submucosal ganglia for agrin (C″) exhibited signs of postmortal decay (e.g., loss of cellular integrity in the epithelium) making the clear localization of agrin uncertain. Nevertheless, the expression pattern of perlecan (A⁗) and COL18A1 (B⁗) was readily detectable at the crypt base and along the crypts. Cell nuclei were counterstained with DAPI. Notably, autofluorescent particles, particularly lipofuscin in enteric neurons was visible throughout the section (arrowheads). Scale: A–C: 200 μm; A′–A″″, B′–B″″, C′–C″″: 20 μm.

**Figure S4:** A comparison of the expression intensities of secretory HSPGs throughout norma‐, hypo‐, and aganglionic segments. The overview (A–C) of entire swiss‐rolled resectates including normo‐, hypo‐, and aganglionic gut regions revealed that the overall intensity of the immunoreactivity of the secretory HSPGs did not change along the gut. The individual segments are shown in the detailed images (normoganglionic A′–C′, hypoganglionic A″–C″ and aganglionic A‴–C‴). Scale: A–C: 2000 μm. A′–C‴: 200 μm.

**Figure S5:** Side‐by‐side comparison of secretory HSPGs at the intermuscular layer between normo‐, hypo‐ and aganglionic segments. The figure shows the expression of perlecan, COL18A1, and agrin at the junction of the two muscle layers in normo‐, hypo‐ and aganglionic gut regions of HSCR‐samples. For better comparison, images were selected which only show the junctions between the muscle layers (i.e., between ganglia). Accordingly, only parts of ganglia in the normoganglionic segment are shown. Perlecan exhibited a strong signal surrounding individual muscle cells of the *Tunica muscularis*, forming a honeycomb pattern which was also detectable in hypo‐ and aganglionic intestinal segments (A). In contrast to perlecan, we did not detect a clearly defined signal of COL18A1 in the *Tunica muscularis* (B). In contrast, agrin was detectable in the surrounding matrix of smooth muscle cells of the *Tunica muscularis*, but less crisp compared to perlecan (C). While Perlecan, COL18A1 and agrin were not detectable between the muscle layers (i.e., the intermuscular layer) in the aganglionic segment, the overall signal intensities of the three secretory HSPGs remained largely constant from normo‐ to aganglionic in the musculature of the *Tunica muscularis*. Scales: A–C 20 μm.

**Figure S6:** Negative controls. The micrographs show a representative section of a HSCR‐sample stained with secondary antibodies only (i.e., no primary antibody). (A) Shows an overview of the entire gut wall, A′–A‴ show high‐power magnification inserts as indicated. Cell nuclei were stained with DAPI. Scales: A 200 μm; A′–A‴ 50 μm.

## Data Availability

The data that support the findings of this study are available from the corresponding author upon reasonable request.
