# Supplementary figures and images for: Perlecan, CollagenXVIII, and Agrin Expression in Normo‐, Hypo‐, and Aganglionic Segments in Hirschsprung's Disease
Source: Neurogastroenterol Motil. 2026 Jan 19;38(1):e70230. doi: 10.1111/nmo.70230 (PMC12816820; doi:10.1111/nmo.70230)

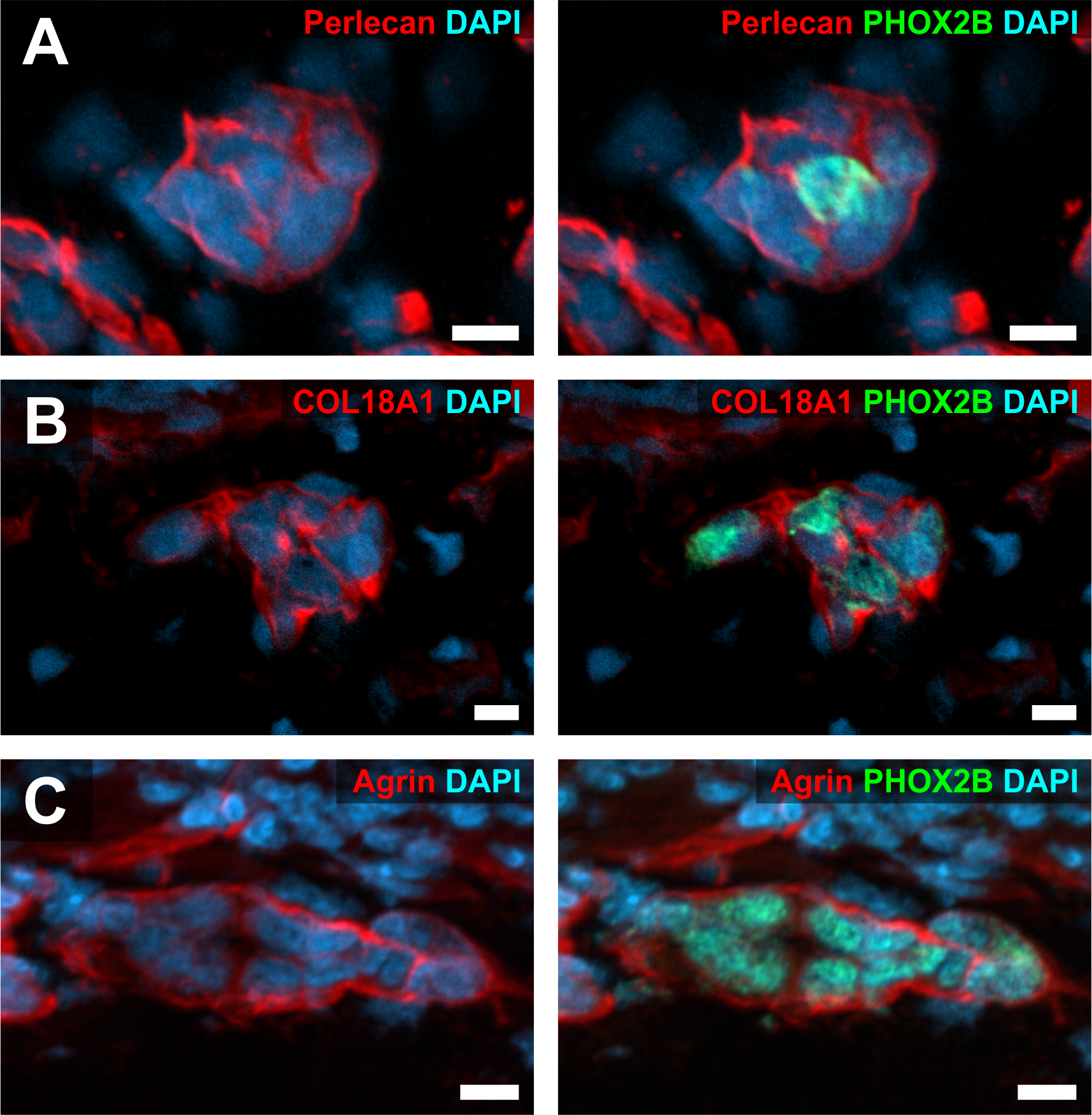

Supplement: Supplementary file 1 — Figure S1: Representative submucosal ganglia in the normoganglionic segment. The micrographs show immunostainings for perlecan (A), COL18A1 (B), and agrin (C) in submucosal ganglia of the normoganglionic segment in HSCR‐patients. Neural cells were counterstained with PHOX2B, cell nuclei were stained with DAPI. Scale: 10 μm. [file NMO-38-e70230-s005.tif]

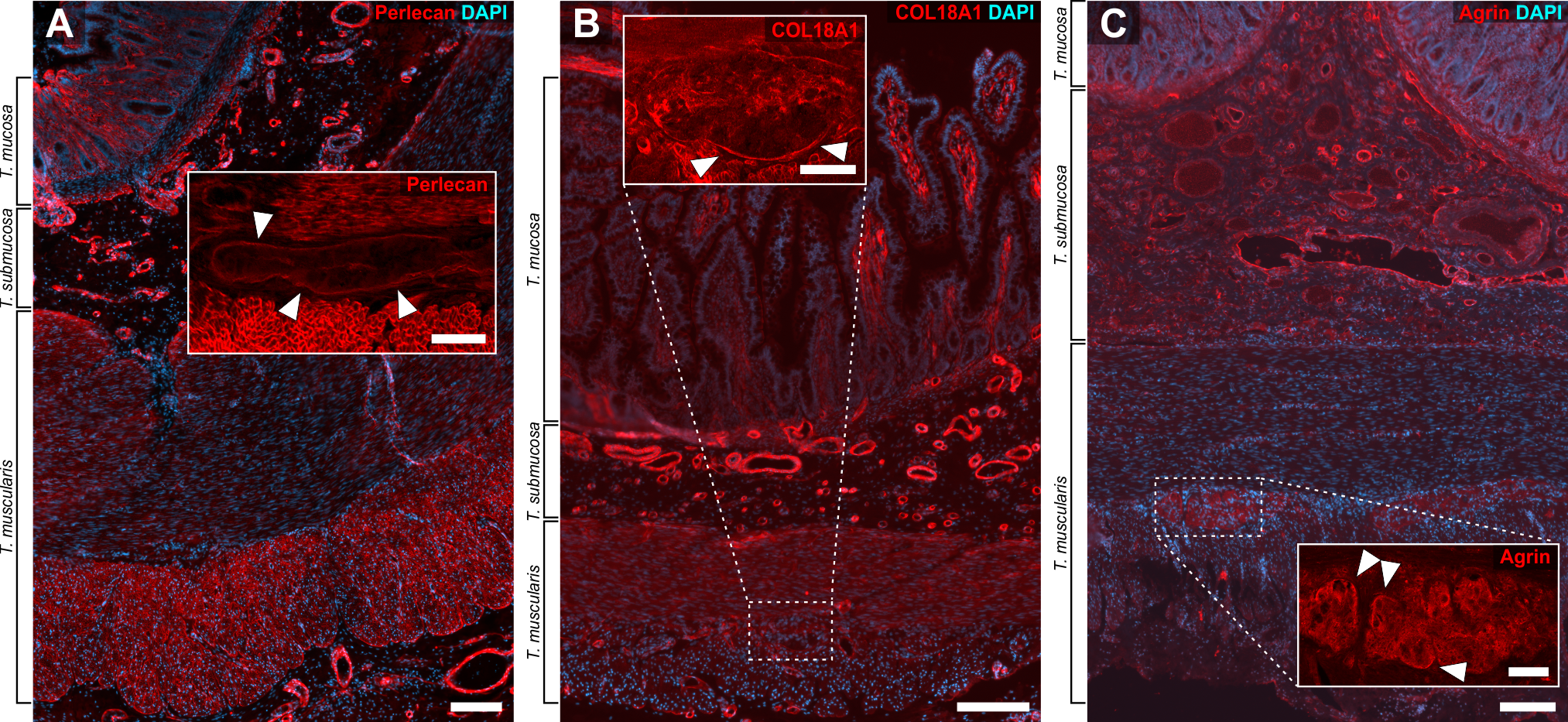

Supplement: Supplementary file 2 — Figure S2: Representative transversal sections of human non‐HSCR control small intestinal segments with perlecan, COL18A1, and agrin staining. The overviews show the entire gut wall immunostained for perlecan (A), COL18A1 (B), and agrin (C). The expressions of perlecan, COL18A1, and agrin were visible at the crypt base and along the crypts, as well as in the surrounding matrix of blood vessels. Furthermore, perlecan and agrin were detectable in the surrounding matrix of smooth muscle cells of the Tunica muscularis, which was not the case for COL18A1. The inserts in A–C are high power magnifications of myenteric ganglia showing that a fine layer of perlecan, COL18A1, and agrin can be seen surrounding the enteric ganglia resembling a basement membrane (arrowheads). Of note, the insert in A is taken from a subsequent section than the overview. Cell nuclei were counterstained with DAPI. Scale: A–C: 200 μm; inserts: 50 μm. [file NMO-38-e70230-s006.tif]

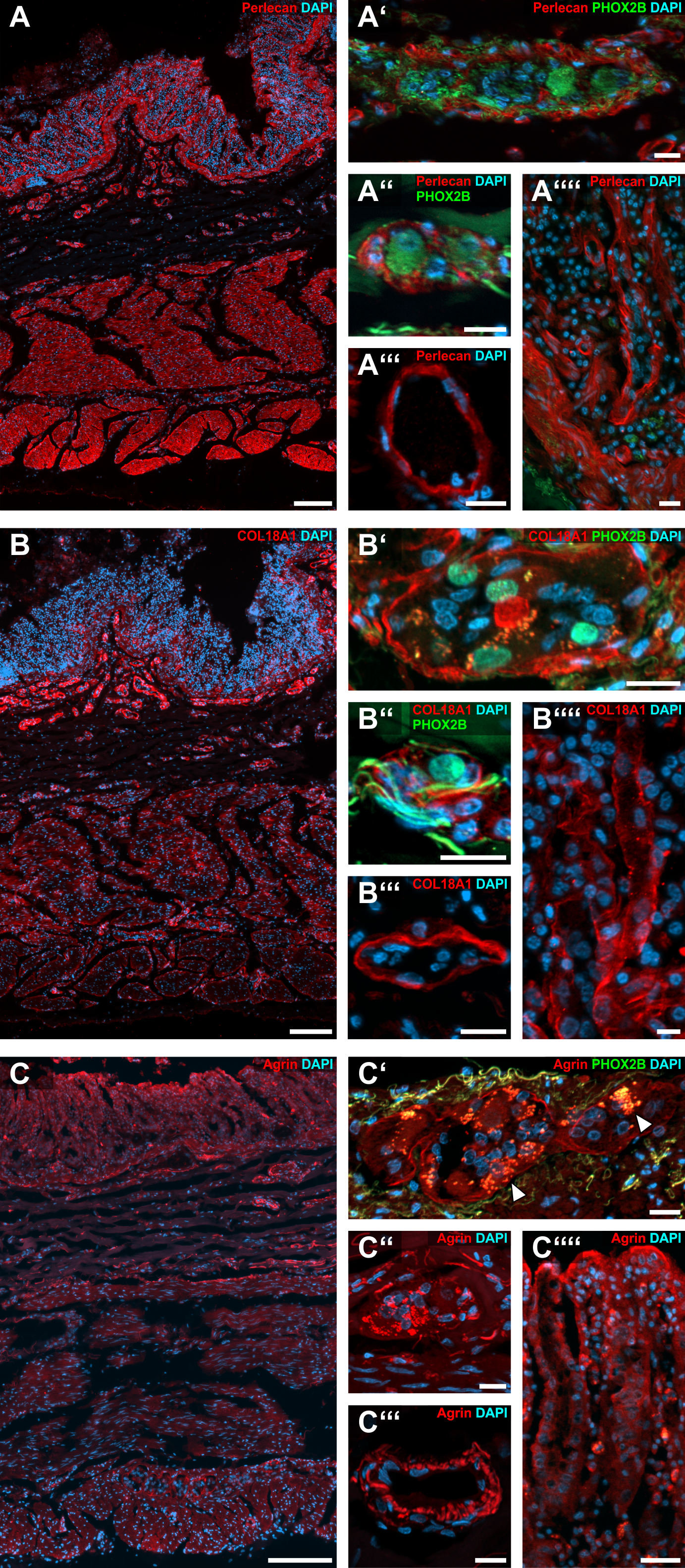

Supplement: Supplementary file 3 — Figure S3: HSPG expression in human non‐HSCR postmortem tissue. Depicted are overviews of the entire gut wall immunostained for perlecan (A), COL18A1 (B) and agrin (C). The high‐power magnification micrographs show myenteric ganglia (A'–C′), submucosal ganglia (A″–C″), blood vessels (A‴–C‴), and the mucosa (A⁗–C⁗). Comparable to pediatric samples, immunoreactivity of perlecan (A′), COL18A1 (B′), and agrin (C′) was detectable surrounding the myenteric ganglia. However, particularly stainings of the mucosal epithelium and submucosal ganglia for agrin (C″) exhibited signs of postmortal decay (e.g., loss of cellular integrity in the epithelium) making the clear localization of agrin uncertain. Nevertheless, the expression pattern of perlecan (A⁗) and COL18A1 (B⁗) was readily detectable at the crypt base and along the crypts. Cell nuclei were counterstained with DAPI. Notably, autofluorescent particles, particularly lipofuscin in enteric neurons was visible throughout the section (arrowheads). Scale: A–C: 200 μm; A′–A″″, B′–B″″, C′–C″″: 20 μm. [file NMO-38-e70230-s002.tif]

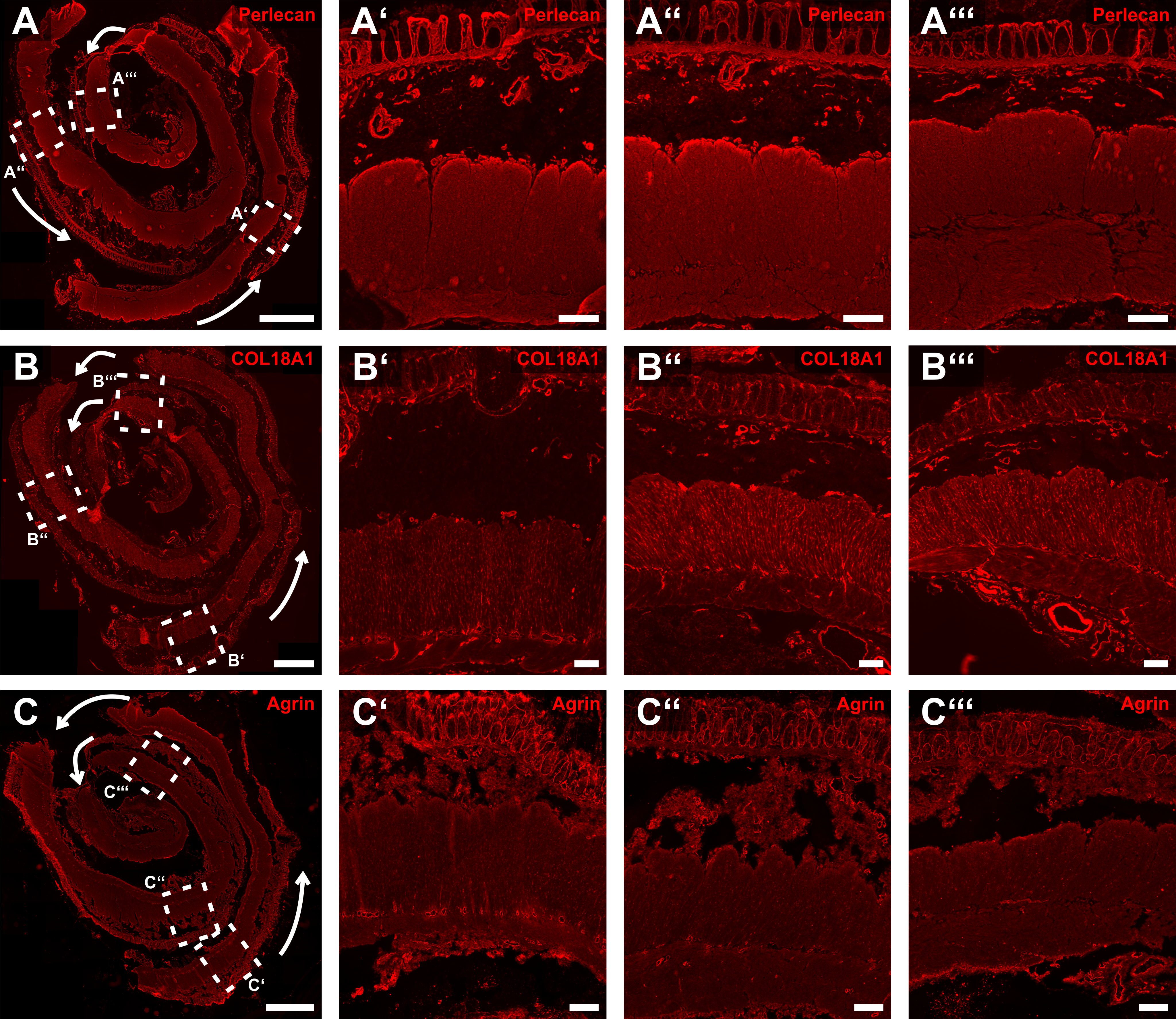

Supplement: Supplementary file 4 — Figure S4: A comparison of the expression intensities of secretory HSPGs throughout norma‐, hypo‐, and aganglionic segments. The overview (A–C) of entire swiss‐rolled resectates including normo‐, hypo‐, and aganglionic gut regions revealed that the overall intensity of the immunoreactivity of the secretory HSPGs did not change along the gut. The individual segments are shown in the detailed images (normoganglionic A′–C′, hypoganglionic A″–C″ and aganglionic A‴–C‴). Scale: A–C: 2000 μm. A′–C‴: 200 μm. [file NMO-38-e70230-s001.tif]

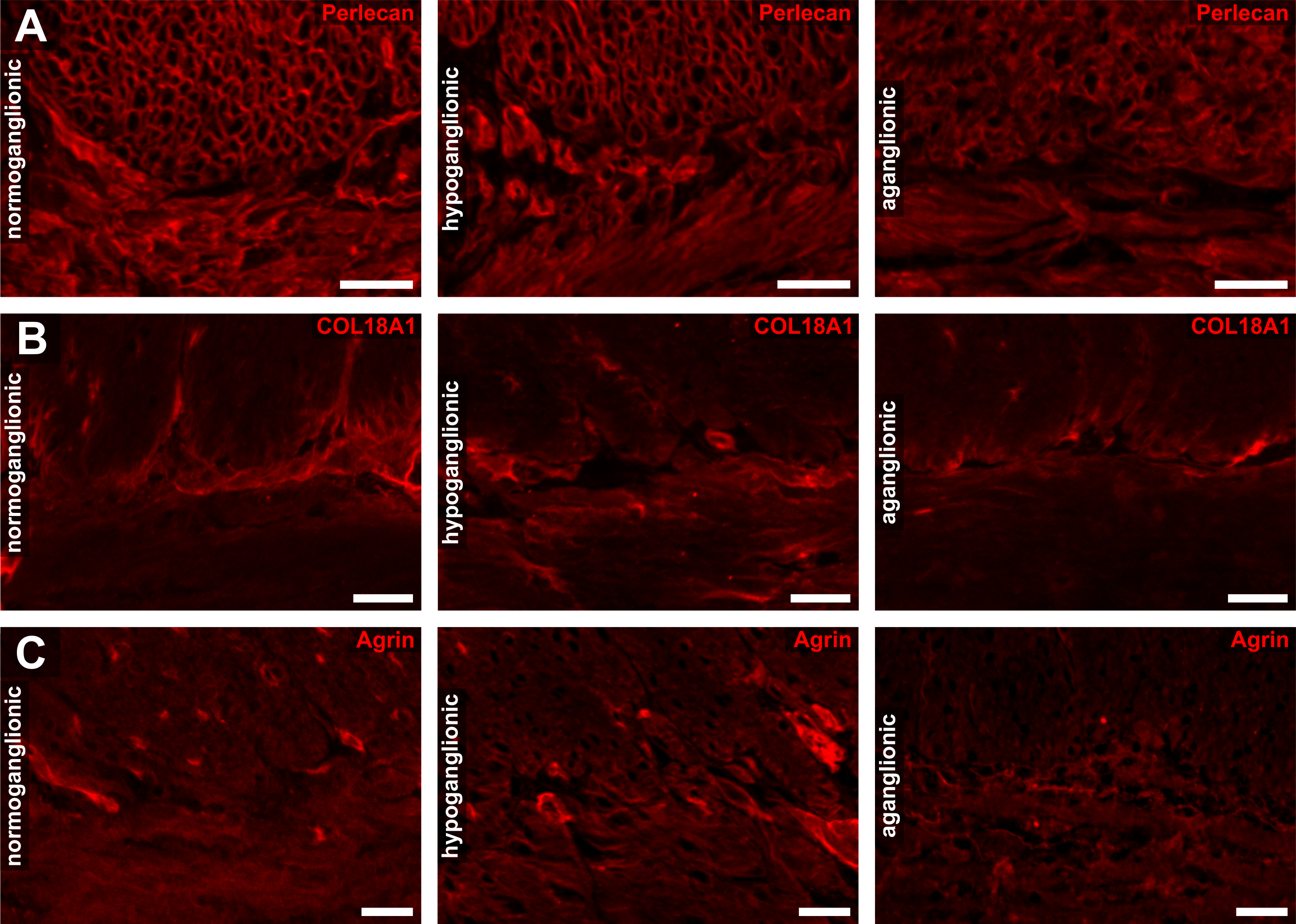

Supplement: Supplementary file 5 — Figure S5: Side‐by‐side comparison of secretory HSPGs at the intermuscular layer between normo‐, hypo‐ and aganglionic segments. The figure shows the expression of perlecan, COL18A1, and agrin at the junction of the two muscle layers in normo‐, hypo‐ and aganglionic gut regions of HSCR‐samples. For better comparison, images were selected which only show the junctions between the muscle layers (i.e., between ganglia). Accordingly, only parts of ganglia in the normoganglionic segment are shown. Perlecan exhibited a strong signal surrounding individual muscle cells of the Tunica muscularis, forming a honeycomb pattern which was also detectable in hypo‐ and aganglionic intestinal segments (A). In contrast to perlecan, we did not detect a clearly defined signal of COL18A1 in the Tunica muscularis (B). In contrast, agrin was detectable in the surrounding matrix of smooth muscle cells of the Tunica muscularis, but less crisp compared to perlecan (C). While Perlecan, COL18A1 and agrin were not detectable between the muscle layers (i.e., the intermuscular layer) in the aganglionic segment, the overall signal intensities of the three secretory HSPGs remained largely constant from normo‐ to aganglionic in the musculature of the Tunica muscularis. Scales: A–C 20 μm. [file NMO-38-e70230-s004.tif]

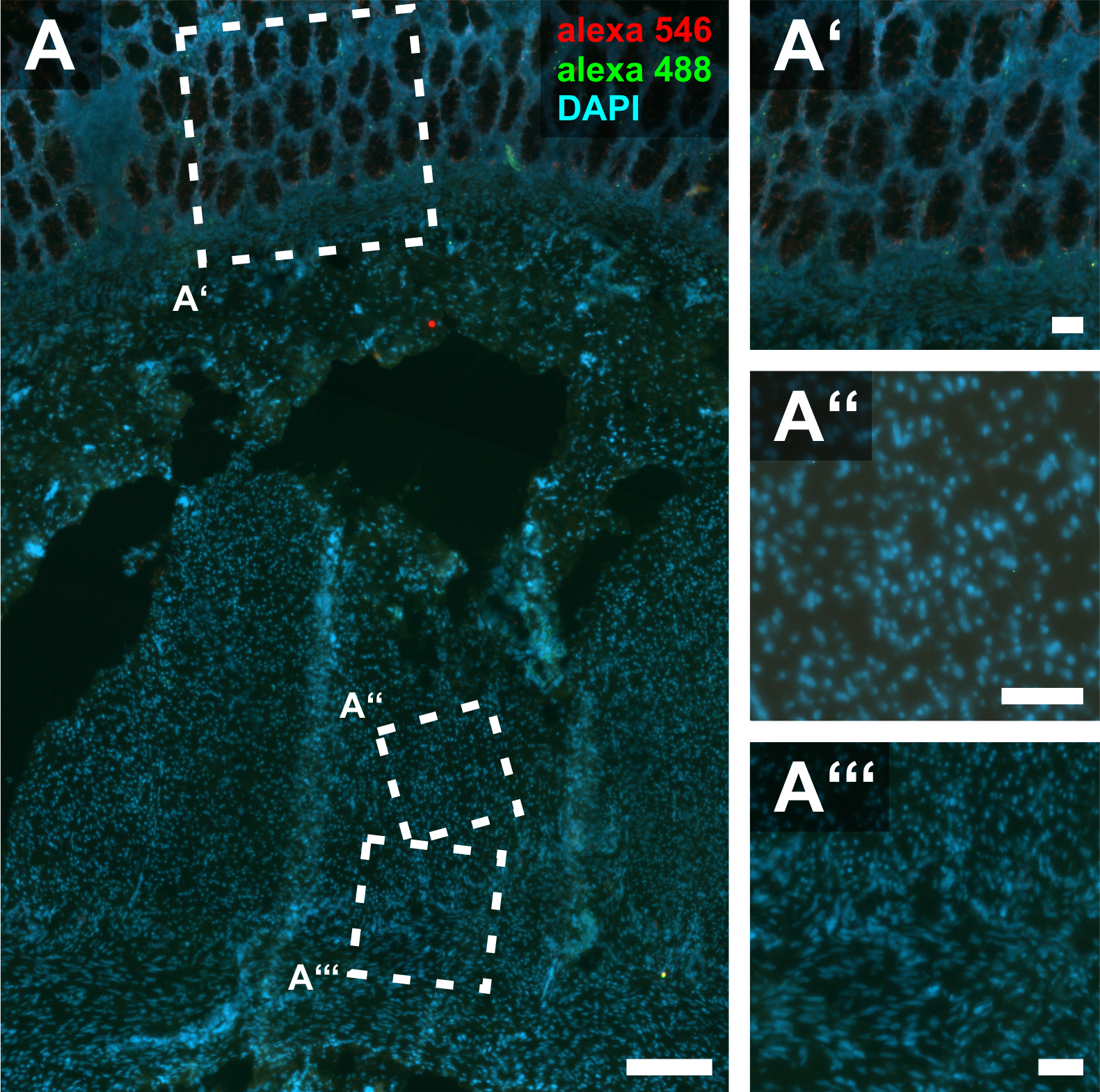

Supplement: Supplementary file 6 — Figure S6: Negative controls. The micrographs show a representative section of a HSCR‐sample stained with secondary antibodies only (i.e., no primary antibody). (A) Shows an overview of the entire gut wall, A′–A‴ show high‐power magnification inserts as indicated. Cell nuclei were stained with DAPI. Scales: A 200 μm; A′–A‴ 50 μm. [file NMO-38-e70230-s003.tif]
